# Structural genomics and the Protein Data Bank

**DOI:** 10.1016/j.jbc.2021.100747

**Published:** 2021-05-03

**Authors:** Karolina Michalska, Andrzej Joachimiak

**Affiliations:** 1 Center for Structural Genomics of Infectious Diseases, University of Chicago, Chicago, Illinois, USA; 2 Structural Biology Center, X-Ray Science Division, Argonne National Laboratory, Lemont, Illinois, USA; 3 Department of Biochemistry and Molecular Biology, University of Chicago, Chicago, Illinois, USA

**Keywords:** structural genomics, structural biology, X-ray crystallography, Protein Data Bank, databases, Hcp, hemolysin-coregulated protein, MCSG, Midwest Center for Structural Genomics, PSI, Protein Structure Initiative, SG, structural genomics, SGC, Structural Genomics Consortium, TSR, thrombospondin type 1 repeat

## Abstract

The field of Structural Genomics arose over the last 3 decades to address a large and rapidly growing divergence between microbial genomic, functional, and structural data. Several international programs took advantage of the vast genomic sequence information and evaluated the feasibility of structure determination for expanded and newly discovered protein families. As a consequence, structural genomics has developed structure-determination pipelines and applied them to a wide range of novel, uncharacterized proteins, often from “microbial dark matter,” and later to proteins from human pathogens. Advances were especially needed in protein production and rapid *de novo* structure solution. The experimental three-dimensional models were promptly made public, facilitating structure determination of other members of the family and helping to understand their molecular and biochemical functions. Improvements in experimental methods and databases resulted in fast progress in molecular and structural biology. The Protein Data Bank structure repository played a central role in the coordination of structural genomics efforts and the structural biology community as a whole. It facilitated development of standards and validation tools essential for maintaining high quality of deposited structural data.

The concept of Structural Genomics (SG) was born as a result of exponential progress in genome sequencing. The fast growth of DNA sequence information in the 1990s led to the generation of huge amounts of genomic data, which was accompanied by significant knowledge gaps in our understanding of biological roles and biochemical functions encoded in the genomes. Of importance, the sequence information bore little insights about the proteins (often called hypothetical) these newly discovered genes programmed, hampering progress toward functional interpretation. Massive accumulation of genomic and metagenomic sequences posed many questions that could not simply be neglected or ignored. To address these new challenges, the National Institutes of Health, Department of Energy, RIKEN, Gates Foundation, Wellcome Trust, and other numerous government and private agencies around the world funded structural genomics programs as early as 1997 to 2000. Table 1 summarizes the contribution of larger SG programs to determination of protein structures.

**Table 1:** Top 20 structural genomics programs

Center	Number of PDB deposits	Origin and funding	Techniques used
RIKEN Structural Genomics/Proteomics Initiative	2746	Japan, government, National Project on Protein Structural and Functional Analyses	NMR, X-ray
Midwest Center for Structural Genomics	1955	USA, PSI/NIH/NIGMS	X-ray, NMR
Structural Genomics Consortium	1896	International/a public–private partnership	X-ray, NMR
Joint Center for Structural Genomics	1601	USA, PSI/NIH/NIGMS	X-ray, NMR
Center for Structural Genomics of Infectious Diseases	1359	USA, NIH/NIAID	X-ray, NMR, cryo-EM
Seattle Structural Genomics Center for Infectious Disease	1355	USA, NIH/NIAID	X-ray, NMR, cryo-EM
Northeast Structural Genomics Consortium	1234	USA, PSI/NIH/NIGMS	X-ray, NMR
New York SGX Research Center for Structural Genomics	1041	USA, PSI/NIH/NIGMS	X-ray, NMR
New York Structural Genomics Research Consortium	364	USA, PSI/NIH/NIGMS	X-ray, NMR
TB Structural Genomics Consortium	344	International worldwide consortium/Various	X-ray, NMR
Center for Eukaryotic Structural Genomics	219	USA, PSI/NIH/NIGMS	X-ray, NMR
Montreal-Kingston Bacterial Structural Genomics Initiative	132	Canada, Canadian Institutes of Health Research	X-ray, NMR
Southeast Collaboratory for Structural Genomics	122	USA, PSI/NIH/NIGMS	X-ray, NMR
Structural Proteomics in Europe	118	European Union	X-ray, NMR
Berkeley Structural Genomics Center	101	USA, PSI/NIH/NIGMS	X-ray
Enzyme Discovery for Natural Product Biosynthesis	91	USA, NIH	X-ray
Structural Genomics of Pathogenic Protozoa Consortium	73	USA, PSI/NIH/NIGMS	X-ray, NMR
New York Consortium on Membrane Protein Structure	70	USA, PSI/NIH/NIGMS	X-ray
Structure 2 Function Project	54	USA, PSI/NIH/NIGMS	X-ray, NMR
GPCR Network	52	USA, PSI/NIH/NIGMS	X-ray

NIAID, National Institute of Allergy and Infectious Diseases; NIGMS, National Institute of General Medical Sciences; NIH, National Institutes of Health; PSI, Protein Structure Initiative.

The mission of SG programs was to facilitate rapid *de novo* structure determination for proteins representing new protein families to provide meaningful structural coverage of the genomes (1, 2, 3), with the presumption that eventually it would be possible to generate good-quality three-dimensional models of all proteins (4). Such a goal could be achieved by structural characterization of representative members of protein sequence families, followed by homology modeling for the remaining proteins. Selection of protein targets for structural studies has therefore become a crucial component of this effort (5, 6, 7, 8, 9), and it remains important today (10). The structural biology research was set to undergo a major transformation.

There were urgent needs and significant challenges to advance technologies for preparation of thousands of proteins and for their structural and functional characterization. The SG programs quickly recognized and attacked deficiencies in protein production and structure solution methods, improved effectiveness and reproducibility of scientific experiments. As a result, in the past 25 years, a number of world-wide structural genomics programs developed high-throughput pipelines for target selection, protein production, characterization, crystallization, and *de novo* structure determination by synchrotron-based X-ray crystallography and NMR (11, 12, 13, 14). These standardized protocols ensured reproducibility of experiments and resulted in higher data quality. The tools developed by the SG consortia that streamlined the gene-to-structure approach significantly benefitted biological and biomedical research, providing insights into novel structural and functional space (11, 15, 16, 17, 18, 19). The advancements resulted in the determination of over 14,000 protein structures worldwide, mostly from unique protein families, and increased structural coverage of the rapidly expanding protein universe. These three-dimensional models based on experimental data were deposited to the macromolecular structure repository, the Protein Data Bank (PDB, (20)), and were made immediately available to the scientific community. Similarly, the advanced technologies that aimed to make structure determination efficient and models more accurate were disseminated broadly and adopted by the biology community. The experimental data generated by the SG centers are freely available to the community and have been utilized by scientists in various fields of research.

By contributing to structural coverage of thousands of protein families (21, 22), SG programs provided many targets for the Critical Assessment of Techniques for Protein Structure Prediction (CASP) (23), a community-wide, biannual experiment to determine the state and progress of protein structure prediction. Characterization of unique structural folds generated training datasets to protein structure prediction algorithms and enormously improved the quality of models in CASP14 (24, 25), getting closer to a major goal of SG programs of obtaining good-quality three-dimensional models for all proteins.

## Structural genomics programs

**Figure 1:**
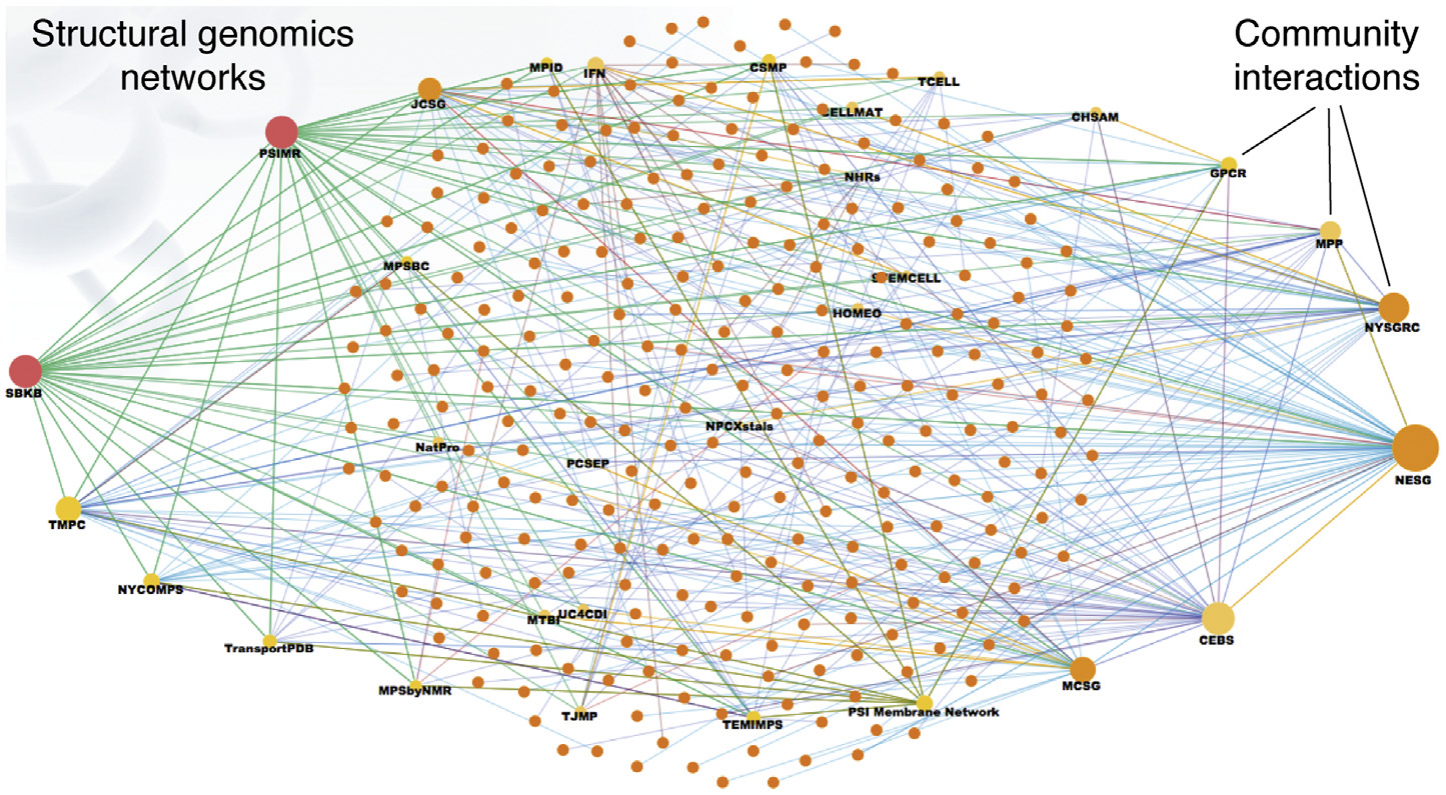
**Structural genomics networks (****http://sbkb.org/metrics/****).** The *dots* represent community interactions.

**Figure 2:**
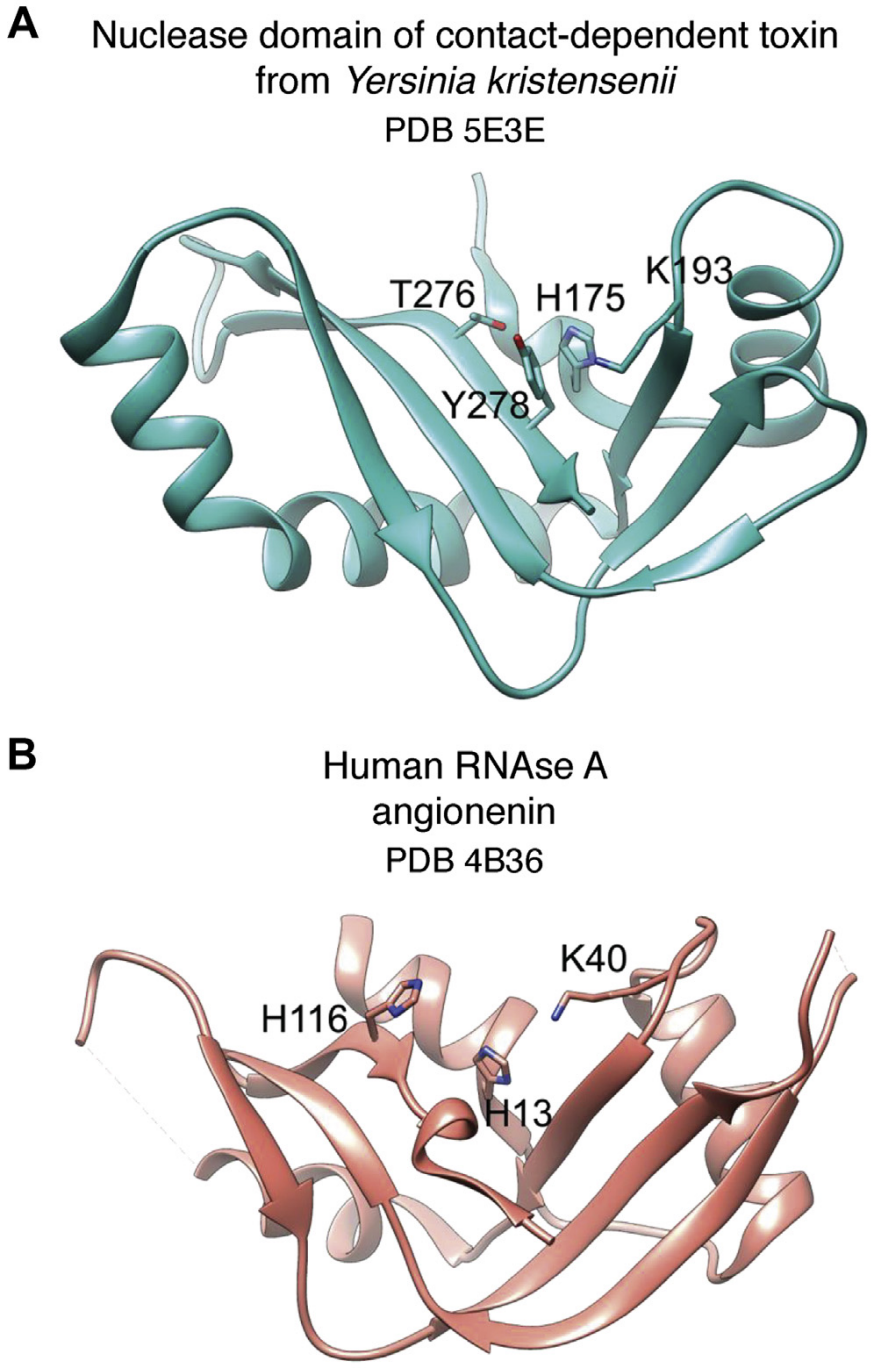
**Discovery of a member of RNase A family in bacteria that serves as a toxin in contact-dependent growth inhibition** (27) **serves as a good example of structure solved by the Midwest Center for Structural Genomics in partnership with biology community.***A*, nuclease domain of contact-dependent toxin from *Yersinia kristensenii* (PDB 5E3E). *B*, human RNase A angiogenin (PDB 4B36) (27).

The US structural genomics effort was launched in 2000, when the National Institutes of Health (NIH) funded the pilot phase of the Protein Structure Initiative (PSI) (http://www.nigms.nih.gov/Initiatives/PSI/). The PSI had three phases. In the first phase (PSI-1), nine centers were established focusing on structural genomics studies of a range of model organisms. During this 5-year period, over 1100 protein structures were determined, more than 700 of which were classified as “unique” owing to their low sequence identity (<30%) with other structurally characterized proteins. In the second phase (PSI-2), the number of funded research centers expanded to include four large-scale “production” centers. The goal was to use methods introduced in PSI-1 to determine a large number of proteins and continue development in streamlining the SG pipelines. By the end of PSI-2, the program had delivered to the community over 4800 protein structures; 85% of these were unique. Many of the structures were of proteins of unknown function. The third PSI phase was called PSI:Biology and intended to increase emphasis on the immediate scientific impact of structures. The PSI centers network worked collaboratively with community investigators and applied the established structure determination pipelines to study a broad range of important biological and biomedical problems, such as complexes and membrane proteins. The SG centers formed extensive interaction and collaboration networks (Fig. 1) that were highly impactful. For example, biology partnership between the Midwest Center for Structural Genomics (MCSG) and the Natural Product Biology Partnership resulted in 68 PDB deposits and 38 peer-reviewed publications (see example (26)). Collaboration within smaller partnerships also led to important contributions, sometimes in novel, emerging fields such as bacterial contact-dependent growth inhibition and signaling. One of these structures showed for the first time that fully functional RNase A–like enzymes are present in bacteria (Fig. 2) (27). By the end of the PSI program, there were more than 9400 structures determined, with the majority of them being unique. Nearly 90% of these were determined by X-ray crystallography, and the rest by NMR (22).

In parallel to the US effort, there were several other structural genomics programs in Canada, Europe, Japan, and China (the Structural Genomics Consortium [SGC]), *Mycobacterium Tuberculosis* Structural Proteomics Project, Europe Structural Proteomics in Europe (SPINE) and others, Protein 3000 implemented in the RIKEN Structural Genomics/Proteomics Initiative (RSGI), and international collaborations International TB Structural Genomics Consortium (TBSGC). The TBSGC focused exclusively on functionally characterized proteins and potential drug targets from *Mycobacterium tuberculosis*.

In 2007, the National Institute for Allergy and Infectious Diseases started a structural genomics program, Structural Genomics Centers for Infectious Diseases, targeting the emerging and re-emerging (drug-resistant) human pathogens. The program established two centers and emphasized target submissions from the wider biology community. These two centers determined, thus far, over 2700 structures, more than 50% of these structures were community-nominated targets.

The importance of developing high-throughput methods became very evident when the COVID-19 pandemic emerged, and we needed to obtain structural information about SARS-CoV-2 proteins to assist drug and vaccine development. In striking contrast to SARS-CoV international effort that from 2003 to 2007 generated ~20 structures, since the emergence of the SARS-CoV-2, the scientific community has contributed over 1200 structures (28), with ~10% of them determined by two Structural Genomics Centers for Infectious Diseases centers. Most of these structures were determined by X-ray crystallography (for example, (29, 30, 31, 32, 33)), but there was very impressive and important contribution from cryo-EM as well (28, 33, 34).

## Highlights of the SG accomplishments

**Figure 3:**
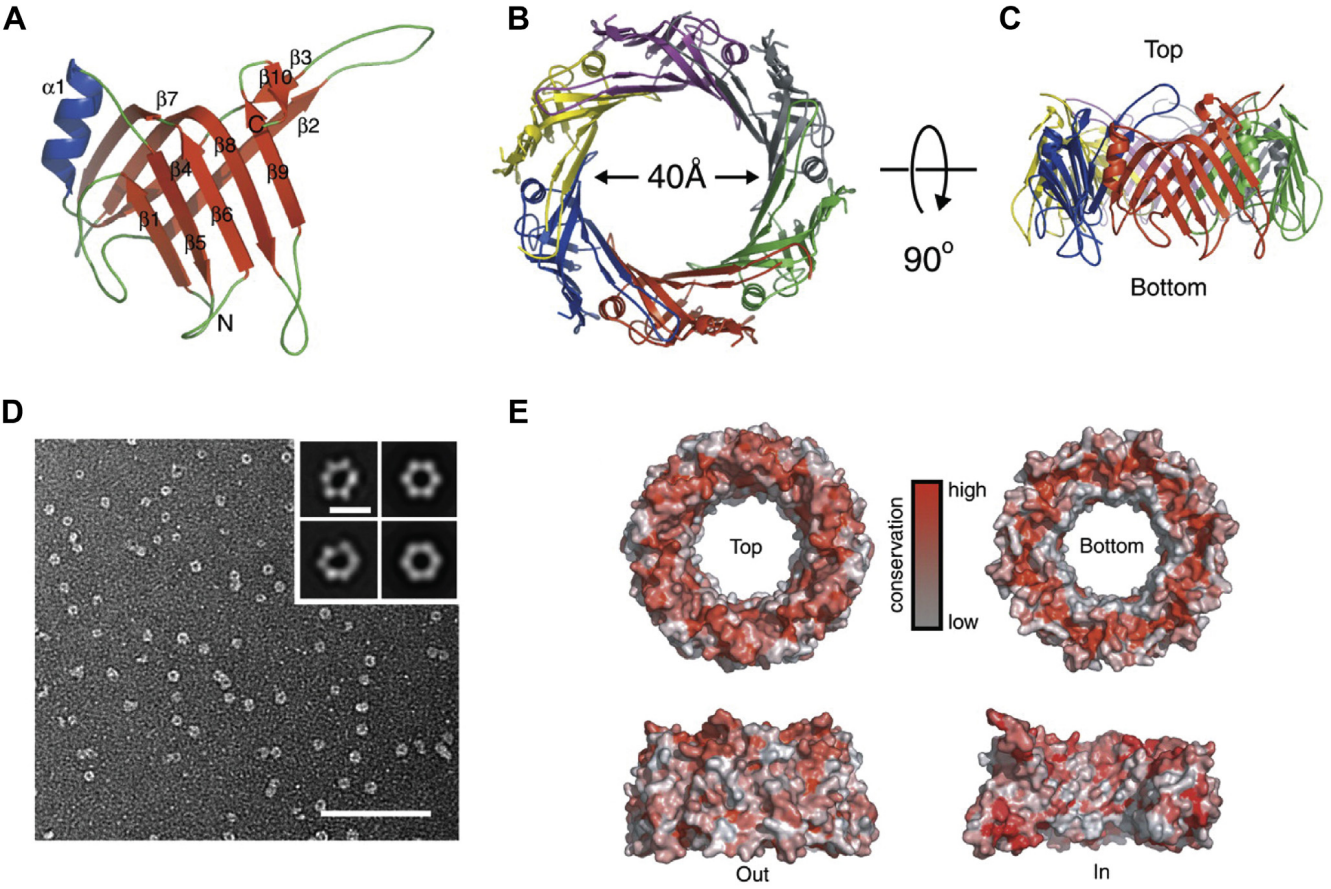
**Structure of Hcp1 protein.** Hsp1 forms a hexameric ring with a large internal diameter. *A*, *Ribbon* representation of the Hcp1 monomer colored by secondary structure: b strands, *red*; a helices, *blue*; and loops, *green*. *B*, *Top view* of a *ribbon* representation of the crystallographic Hcp1 hexamer. The individual subunits are colored differently to highlight their organization. *C*, edge-on view of the Hcp1 hexamer shown in (*B*). *D*, electron microscopy and single-particle analysis of Hcp1. Electron micrograph of Hcp1 negatively stained with 0.75% (w/v) uranyl formate. Scale bar, 100 nm. *Inset*, *Left*, representative class averages and (*right*) the same averages after 6-fold symmetrization. *Inset* scale bar, 10 nm. *E*, sequence conservation analysis of Hcp1. An alignment of 107 Hcp proteins in 43 Gram-negative bacteria was used to plot the relative degree of conservation at each amino acid on the surface of Hcp1. Conservation is indicated by color, where *red* residues are highly conserved and *white* residues are poorly conserved. Figure from (35).

**Figure 4:**
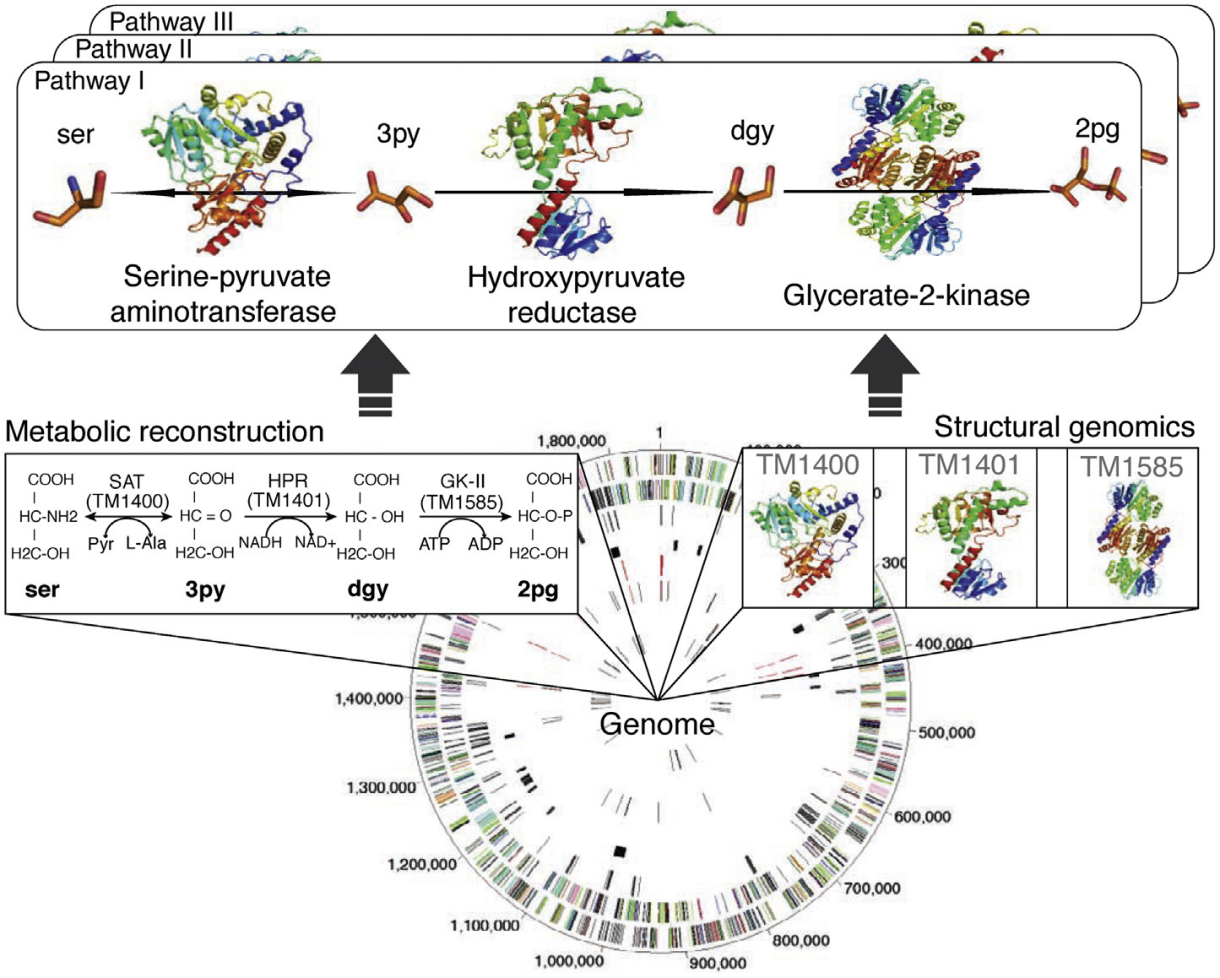
**Combining metabolic reconstruction and structural genomics approaches for an integrated annotation of the *T. maritima* central metabolic network.** Underlying genomics information (*bottom*) enabled both a metabolic reconstruction (*left subpanel*) and an atomic-level structure determination/modeling of all *T. maritima* proteins (*right subpanel*). Integration of these two approaches enabled detailed information to be acquired for every reaction in the network (*upper subpanel*); an example from the *T. maritima* serine degradation pathway is illustrated. Figure taken from (37).

**Figure 5:**
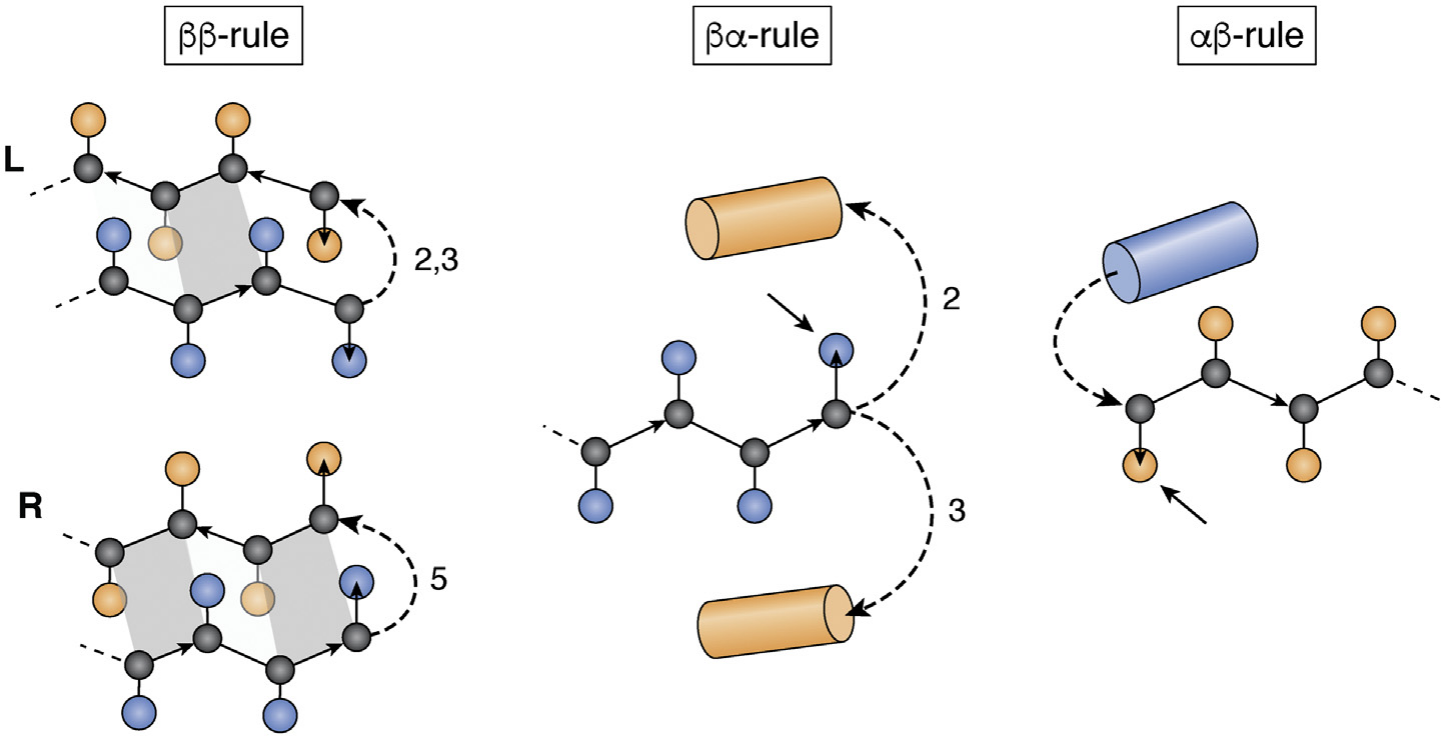
**Fundamental rules of designing proteins relating local backbone structures to favorable tertiary motifs.***Left*, ββ-rule, the chirality of β-hairpins is determined by the length of the connecting loop. The chirality is defined on the basis of the pleat of the strand residue preceding or following the connecting loop. *Middle*, βα-rule, the helix direction is determined by the pleat direction of the last strand residue and the length of the connecting loop. *Right*, αβ-rule, the pleat of the first strand residue points away from the helix (39). Figure provided by Dr Nobuyasu Koga (Institute of Molecular Science, Japan).

**Figure 6:**
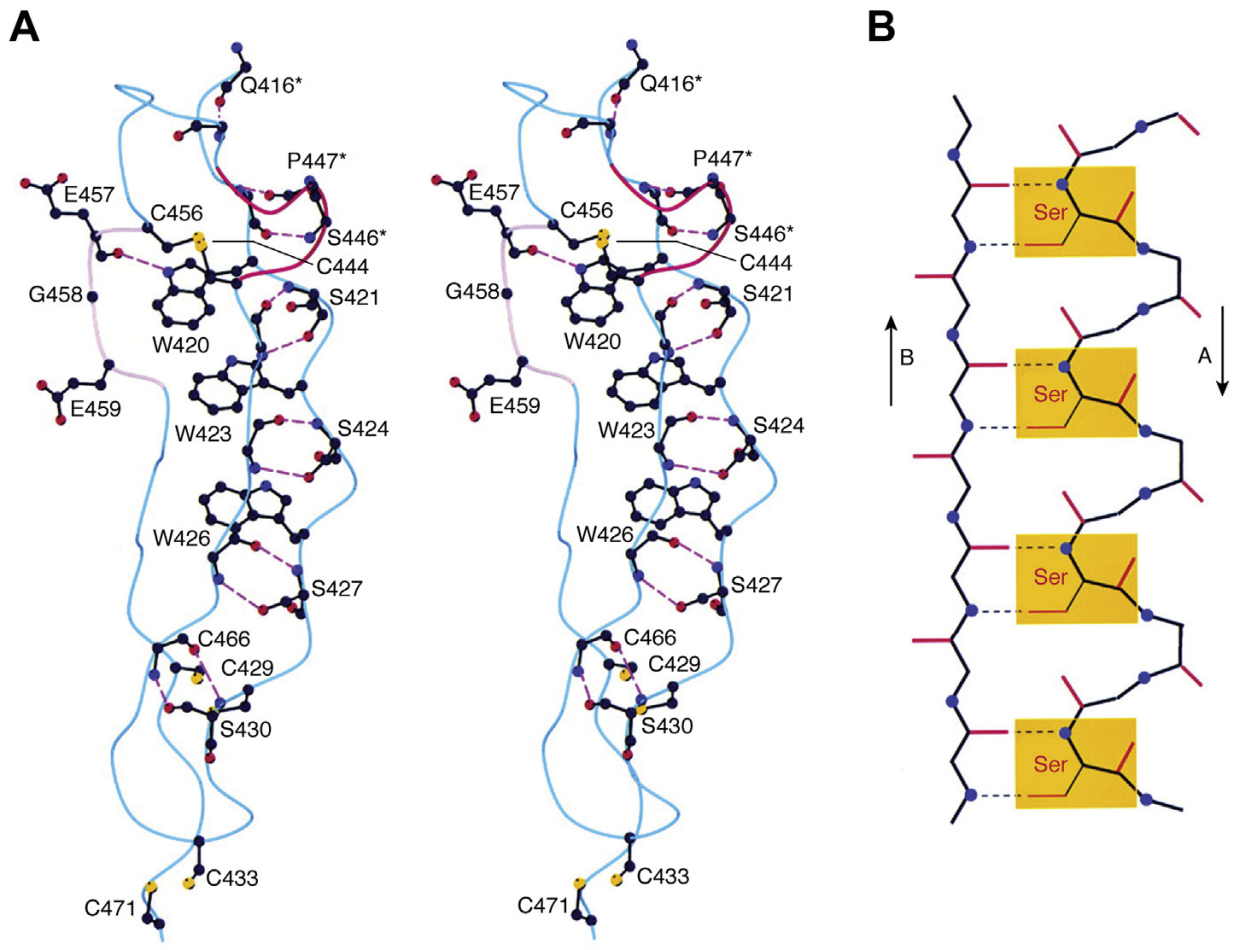
**CWR-layered core structure of the TSR domain.***A*, a stereoview of C, W, and R layers in TSR2 of TSP-1. Displayed residues that are directly involved in forming the layered structure are drawn in *ball* and *stick* representation with salt bridges, and hydrogen bonds drawn as *dashed lines*. The big jar handle motif, which is associated with the first W layer is highlighted in *pink*. *B*, a schematic drawing of the CWR-layered structure with each layer and layer-forming residue(s) labeled. The residue Glu459 that is marked with an *asterisk* forms a hydrogen bond between its main chain carbonyl group and the side chain of Arg442 in the R1 layer. The three antiparallel strands are drawn in lines schematically with *arrowheads* indicating their polarities. The three bulges associated with the rippled strand A and the big jar handle are also shown. Figure taken from (40). TSR, thrombospondin type 1 repeat.

SG programs produced a number of high-profile results in collaboration with the biology community. Here we show several examples from PSI centers. The MCSG determined several structures of hemolysin-coregulated protein (Hcp). These proteins are highly conserved among Gram-negative proteobacteria and were suspected to be part of the type VI secretion apparatus. They shared little sequence homology with proteins of known structure. In an effort to gain insight into the function of these proteins, the crystal structure of Hsp1 from *Pseudomonas aeruginosa* was determined (Fig. 3). This Hcp1 protein formed hexameric rings that can stack and create a wide channel used for protein secretion (35). Later, the MCSG determined a structure of Hsp3, a low-sequence identity Hsp1 paralog from *P. aeruginosa* that shows a very similar architecture (36). Joint Center for Structural Genomics combined structures available in the PDB (several of which were determined by PSI centers) with homology models and, for the first time, generated a three-dimensional reconstruction of metabolic networks in the bacterium *Thermatoga maritima* (Fig. 4) (37). The Joint Center for Structural Genomics has showed that one can integrate structural data with networks analysis to inform about functions, mechanisms, and evolution of cellular systems. Another PSI center, the New York SGX Research Center for Structural Genomics, systematically studied structures of protein phosphatases from human and biomedically relevant pathogens, including *Toxoplasma gondii*, *Trypanosoma brucei*, and *Anopheles gambiae*. These enzymes are important drug targets, and their crystal structures provide insights into regulation, signaling, and development processes. Together with the contributions from other SG consortia, it allowed to build a database and materials repository for structure-guided experimental and computational drug discovery for protein phosphatases (38). Northeast Structural Genomics Consortium funded by PSI contributed important data to understand the rules of protein structures and helped developing tools for protein design (39). These rules relate secondary structural patterns to protein tertiary motifs (Fig. 5). Based on these guidelines it was possible to engineer a stable, funnel-shaped protein fold. The SG programs determined many novel structures including those with new folds. One example is shown in Figure 6 (40). Thrombospondin type 1 repeats (TSRs) showed a novel, antiparallel, three-stranded fold that consists of alternating stacked layers of tryptophan and arginine residues and is capped with disulfide bonds on each end. The structure of the TSR domain provides insight into structural and functional studies of the TSR superfamily. TSRs play a role in mediating cell attachment, glycosaminoglycan binding, and inhibition of angiogenesis and matrix metalloproteinases.

## Databases and repositories

During the initial trial period it was shown that it is possible to establish high-throughput semiautomated production pipelines and generate large number of proteins in quantities suitable for structural studies. It also became clear that the success rate of these pipelines was not very high, exposing the necessity to collect all generated information and analyze the data to improve target selection, technologies, and protocols (41). Therefore, software and database developments were necessary to handle high-throughput structure determination workflows and, overall, they have led to production of better proteins for structural biology, structures of higher quality and improved integrity of the associated data. To further disseminate structural genomics materials, the Material Repository (PSI-MR) (42) was created to store and distribute biological reagents, primarily expression clones at low cost.

Databases were developed to track trials and improve effectiveness and reproducibility of experiments. These were first created as local resources that later were combined into centralized databases (22, 43), with the final coordinates and structure factors files reaching to the PDB. SG-created resources included Target Registration Database (TargetDB) (44, 45) and PepcDB (Protein Expression Purification and Crystallization Data Base; (46)), which were eventually merged in the TargetTrack knowledgebase (47) and Structural Biology Knowledgebase (41, 48). These databases exposed limitations of existing resources; for example, files deposited to the PDB were missing important information about projects because including these data in deposition was optional. Clearly, the SG structures presented new challenges to the PDB (49). These programs were also very different because of the National Institutes of Health requirements to make all generated data available to the community. The original guidelines for deposition were established in 1989 as part of the International Union for Crystallography initiative. Validation standards were later set as part of a wwPDB project in which Task Forces made recommendations and the wwPDB implemented them (50, 51, 52). The SG programs and biology community worked together with the PDB to facilitate the rapid deposition of data and track the progress of the work. At the same time, the American Crystallography Association created committees to formulate guidelines for structure deposition. In a series of workshops and extensive discussions, standards were established for X-ray crystallography deposits and later for NMR and cryo-EM structures as well (53, 54, 55, 56, 57). A set of PDB deposition guidelines was published and subsequently adopted by funding agencies and scientific journals (52). Today, they are broadly implemented and serve as an example to the entire scientific community. Structural genomic programs monitored structure quality, which resulted in overall improvement of deposited structures. The growth of the PDB was incredible. Between 2001, when the first SG structures were deposited, and 2016 when the majority of SG structures were completed, the PDB deposits increased from 2814/year to 10,819/year, or 3.84 times, with SG programs contributing significant fraction of unique structures.

## Current status and future outlook

Today the PDB offers online tools, summary reports, protein sequence information and redundancy, other data associated with protein structure determination, and links to homology models (46). Functional coverage can be examined according to enzyme classification, gene ontology (biological process, cell component, and molecular function), and disease (58).

Structural genomics projects propelled technology development and helped to disseminate it through the biology community. Structure solution using X-ray diffraction at light sources was never simpler. The tools developed for structure validation help to rapidly identify potential issues and guide improvement of structural models. The PDB has become a fully integrated, single global repository of experimentally determined 3D structures of biological macromolecules and their complexes, which the community can access and analyze the structural data (59, 60). Archives for homology models (61) and integrative/hybrid structures are available (62). Raw data can be deposited into versatile servers (63, 64), although challenges remain as the amount of data increases exponentially with serial crystallography experiments collected at FELs and other light sources (65). There are ongoing discussions to better integrate with other databases and new community resources, especially in support of drug discovery (66), rapidly expanding cryo-EM data (67), deep learning models (68), as well as Department of Energy funded Systems Biology Knowledgebase, KBase (69) and others.

## Dedications

Dedicated to Professor Wladek Minor on the occasion of his 75th birthday.

## Conflict of interest

The authors declare that they have no conflicts of interest with the contents of this article.
